# The Military Cross for Medical Heroism

**Published:** 1915-07-10

**Authors:** 


### THE MILITARY CROSS FOR MEDICAL HEROISM.

### Decorations for Colonial Doctors.

In a Supplement to the *London Gazette*, which was issued on Saturday last, it is announced that the King has conferred the Military Cross on the following medical officers in recognition of their gallantry and devotion to duty whilst serving with the Expeditionary Force:—

Tempy. Lieut. John Marchbank Gillispie, M.B., R.A.M.C.

On May 24 and 25, 1915, at Ypres, he displayed conspicuous gallantry in ministering to the wounded under fire. He traversed the ground many times while under heavy shell and rifle fire, and dressed the wounded in the open. On the night of May 25 he went up to a wood near Bellegarde Farm, and searched for wounded men close up to the German trenches.

In every action his gallantry has been conspicuous.

Tempy. Lieut. John Hart McNichol, M.B., R.A.M.C.

On May 24 and 25, 1915, at Ypres, with untiring energy and gallantry attended to wounded men under heavy rifle and shell fire, saving the lives of many men.

On the night of May 25 he searched a wood near Bellegarde for the wounded, attended to them, and had them brought in. This wood was close up to the German trenches.

He has shown the greatest courage in attending to the wounded in action.

Assistant-Surgeon Edwin Bunkall Messinier, Indian Subordinate Medical Department.

For consistent good work, gallant conduct, and devotion to duty when X Battery, Royal Horse Artillery, was in action on May 9 and 10, 1915. He went under shell fire to assist the wounded, and, although twice wounded, continued to perform his duties after having his wounds dressed.

### COLONIAL MEDICAL HEROISM.

In connection with the notification which was published in the *London Gazette* of June 3, the acts of gallantry and distinguished service at the Dardanelles, for which the Distinguished Service Order and the Military Cross respectively were awarded, include the following performed by medical men, with the decoration awarded:—

Major Eugene Joseph O'Neill, F.R.C.S., New Zealand Medical Corps.

On April 25 and 26, 1915, during operations near Gaba Tepe, for exceptionally good service and exhibiting initiative and resource in command of a bearer subdivision.

Captain Arthur Graham Butler, Australian Army Medical Corps (attached 9th Australian Infantry Battalion).

During operations in the neighbourhood of Gaba Tepe on April 25, 1915, and subsequent dates, for conspicuous gallantry and devotion to duty in attending wounded under heavy fire, continuously displaying courage of a high order.

